# Vectorcardiography reflects left ventricular reverse remodelling after cardiac resynchronization therapy for both left bundle branch area pacing and biventricular pacing

**DOI:** 10.1093/europace/euag203

**Published:** 2026-08-04

**Authors:** Karin C Smits, Florien Klein, Lisette, M Neyari, Edoardo Bressi, Domenico Grieco, Roel Meiburg, Julien Garnier, Jesse H J Rijks, Antonius M W van Stipdonk, Justin G L M Luermans, Leonard M Rademakers, Kevin Vernooy, Frits W Prinzen, Uyên Châu Nguyên

**Affiliations:** Department of Physiology, Maastricht University Medical Center+ (MUMC+), Cardiovascular Research Institute Maastricht (CARIM), P. Debyelaan 25, Maastricht 6229 HX, The Netherlands; Department of Cardiology, MUMC+, CARIM, P. Debyelaan 25, Maastricht 6229 HX, The Netherlands; Department of Cardiology, Catharina Ziekenhuis, Eindhoven 5623 EJ, The Netherlands; Department of Cardiology, MUMC+, CARIM, P. Debyelaan 25, Maastricht 6229 HX, The Netherlands; Department of Cardiology, Policlinico Casilino, Rome 00169, Italy; Department of Cardiology, Policlinico Casilino, Rome 00169, Italy; Department of Biomedical Engineering, Eindhoven University of Technology, Eindhoven 5612 AZ, The Netherlands; Department of Cardiology, MUMC+, CARIM, P. Debyelaan 25, Maastricht 6229 HX, The Netherlands; Department of Cardiology, MUMC+, CARIM, P. Debyelaan 25, Maastricht 6229 HX, The Netherlands; Department of Cardiology, MUMC+, CARIM, P. Debyelaan 25, Maastricht 6229 HX, The Netherlands; Department of Cardiology, MUMC+, CARIM, P. Debyelaan 25, Maastricht 6229 HX, The Netherlands; Department of Cardiology, Catharina Ziekenhuis, Eindhoven 5623 EJ, The Netherlands; Department of Cardiology, MUMC+, CARIM, P. Debyelaan 25, Maastricht 6229 HX, The Netherlands; Department of Physiology, Maastricht University Medical Center+ (MUMC+), Cardiovascular Research Institute Maastricht (CARIM), P. Debyelaan 25, Maastricht 6229 HX, The Netherlands; Department of Cardiology, MUMC+, CARIM, P. Debyelaan 25, Maastricht 6229 HX, The Netherlands

**Keywords:** Cardiac resynchronization therapy, Biventricular pacing, Left bundle branch area pacing, Vectorcardiography

## Introduction

Left bundle branch area pacing (LBBAP) has emerged as a promising alternative to biventricular pacing (BiVP.^1,2^ Vectorcardiography (VCG) characterizes electrical activation and repolarization in three dimensions, incorporating both magnitude and direction.^3,4^ VCG parameters have been associated with clinical outcomes following BiVP, with QRS area particularly outperforming conventional QRS duration and morphology.^5–8^ However, the effect of CRT, particularly LBBAP, on VCG changes remains largely unexplored. The aim of this study was to assess the impact of CRT with LBBAP or BiVP on VCG parameters and their relationship with LV reverse remodeling.

## Methods

This was an observational multicenter cohort study. CRT patients receiving LBBAP or BiVP. The study adhered to the Declaration of Helsinki. Patients underwent LBBAP or BiVP at the physician’s discretion in accordance with standard techniques. During LBBAP, left bundle branch capture was confirmed intra-procedurally by the criteria adopted within the MELOS framework.^2^ CRT response was defined as an improvement in LVEF of ≥5% after 6 months follow-up compared with baseline echocardiography.

Standard 12-lead electrograms (ECGs) acquired prior to CRT implantation and after 6 months follow-up were collected. The VCG was reconstructed from the digital 12-lead ECG with the Kors conversion matrix,^9^ using MATLAB R2022a (MathWorks, Natick, MA, USA). The resulting three-dimensional orthogonal leads were used to semi-automatically determine QRS area and T-wave area.^10^ Besides QRS duration and QRS area, QTc was included as an exploratory comparator to test whether repolarization heterogeneity induced by biventricular pacing carries independent predictive value for LV reverse remodelling.

Statistical analyses were performed using IBM SPSS Statistics 28 (IBM Corp., Armonk, NY, USA) and MATLAB R2022a. Between-group comparisons were performed using parametric or non-parametric tests, as appropriate. Within-group changes were assessed using paired tests. Logistic regression was performed to identify parameters associated with LV reverse remodelling.

## Results

A total of 116 CRT patients receiving BiVP (*n* = 66) or LBBAP (*n* = 50) were included, with a mean age of 68 ± 9.1 years, and 71% were male. Patients predominantly had a Class IA indication for CRT (mean baseline LVEF 31 ± 7.8%, QRS duration 184 ± 22 ms, and LBBB 86%). There were no significant differences observed for sex, age, aetiology, NYHA class, LVEF, QRS duration, or LBBB morphology between patients receiving LBBAP and BiVP.

### Vectorcardiographic and electrocardiographic analyses

Both LBBAP and BiVP reduced QRS area (LBBAP: Baseline 107 ± 48, follow-up 55 ± 25, mean *Δ* −52 ± 43 µVs; BiVP: baseline 117 ± 36, follow-up 54 ± 29, mean *Δ* −63 ± 41 µVs), T-wave area (LBBAP: baseline 83 ± 35, follow-up 37 ± 20, mean *Δ* −46 ± 39 µVs; BiVP: baseline 72 ± 27, follow-up 38 ± 18, mean *Δ* −35 ± 33 µVs), and QRS duration (LBBAP: baseline 181 ± 24 ms, follow-up 163 ± 24 ms, mean *Δ* −18 ± 32 ms; BiVP: baseline 187 ± 19 ms, follow-up 158 ± 25 ms, mean *Δ* −28 ± 24 ms). No significant differences in these changes were observed between the LBBAP and BiVP groups. The QTc interval did not change significantly with either LBBAP or BiVP. Additionally, relative changes following CRT were greater for VCG-derived QRS area (−51.6%) and T-wave area (−51.5%) compared with ECG-derived QRS duration (−12.9%) and QTc interval (−2.8%).

A significant positive correlation between ΔQRS area and ΔT-wave area was observed in both the BiVP (*r* = 0.650, *P* < 0.001) and LBBAP groups (*r* = 0.739, *P* < 0.001), with no significant difference in slope between pacing modalities. In contrast, the relationship between ΔQRS duration and ΔQTc interval was weak in the BiVP group (*r* = 0.332, *P* = 0.007) and absent in the LBBAP group (*r* = 0.258, *P* = 0.071).

### Association with left ventricular reverse remodelling

Of all 116 patients, 87 (75%) showed LV reverse remodelling and 29 (25%) did not. Baseline QRS area was not significantly associated with CRT response (OR 1.008, 95% CI 0.998–1.019, *P* = 0.130). However, the change in QRS area following CRT differed significantly between responders and non-responders (median −60.4; IQR −93.8 to −31.6 vs. −31.2; IQR −59.8 to −14.6 µVs, *P* = 0.005).

**Figure 1:**
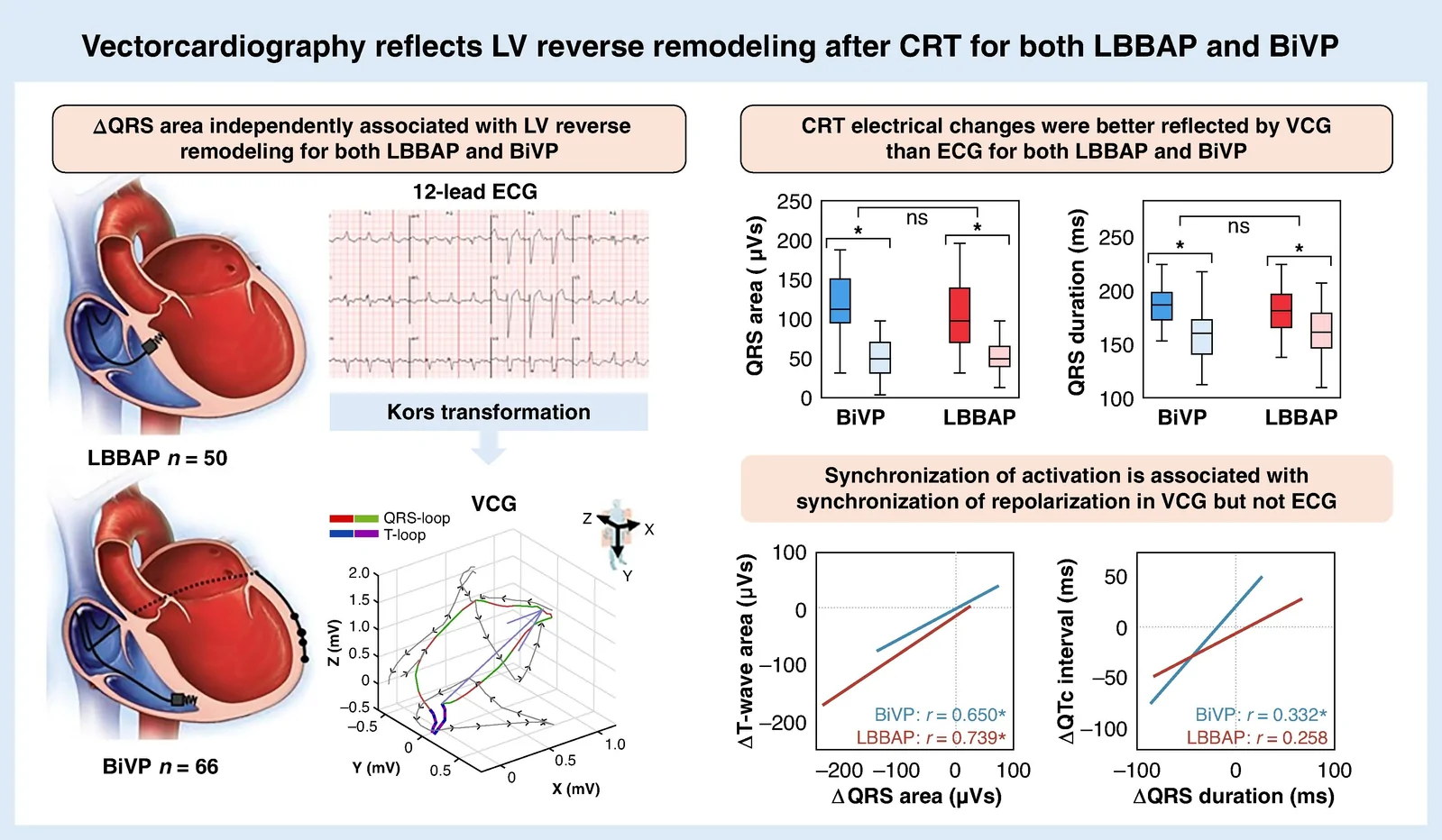
Left panel: reconstruction of VCG from standard 12-lead ECG. In multivariable analyses only reduction in QRS area was independently associated with LV reverse remodelling for both BiVP and LBBAP. Upper right panel: boxplots visualizing QRS area, T-wave area, QRS duration, and QTc interval at baseline and follow-up in patients receiving BiVP or LBBAP. Lower right panel: scatterplots demonstrating the correlation between ΔQRS area and ΔT-wave area and between ΔQRS duration and ΔQTc interval in BiVP and LBBAP. *Statistically significant difference (*P* ≤ 0.05). BiVP, biventricular pacing; ECG: electrocardiography; LBBAP, left bundle branch area pacing; ns: non-significant; *r*: Pearson correlation coefficient; VCG: vectorcardiography.

LVEF increased to a similar extent following LBBAP and BiVP (10 ± 10% vs. 11 ± 11%, *P* = 0.821). In univariable analysis, LBBB and reductions in QRS area and T-wave area were associated with LV reverse remodelling, whereas changes in QRS duration, pacing modality, and QTc interval were not. In multivariable analysis, only the reduction in QRS area remained independently associated with LV reverse remodelling (OR 0.984, 95% CI 0.969–0.999, *P* = 0.041), indicating that VCG-derived parameters, particularly QRS area, more closely reflect CRT-induced electrical changes and its association with LV reverse remodelling than conventional ECG measures. No significant differences were observed between LBBAP and BiVP in the magnitude of changes in QRS area, T-wave area, QRS duration, QTc interval, or LVEF. An overview of these findings are provided in *Figure 1*.

### Limitations

This observational study can only demonstrate associations and does not allow causal inference. Echocardiographic and ECG/VCG analyses were not performed in a blinded core laboratory. However, investigators performing echocardiographic measurements were not involved in implantation procedures or statistical analyses and were therefore effectively blinded to treatment allocation and outcomes. Detailed data on guideline-directed medical therapy, coronary sinus lead position, operator experience, and intracardiac electrogram recordings were not systematically available for all patients.

## Conclusion

In the present study, we investigated changes in VCG parameters in patients undergoing CRT with either LBBAP or BiVP and assessed their association with LV reverse remodelling. The main findings of this study are:

- CRT-induced electrical changes were associated with greater relative changes in VCG-derived parameters than in conventional ECG parameters, for both LBBAP and BiVP.
- Improvements in electrical ventricular activation were accompanied by corresponding changes in repolarization when assessed using VCG, whereas this coupling was not captured by conventional ECG parameters.
- Only reduction in QRS area remained independently associated with LV reverse remodelling.

These findings suggest that VCG-derived parameters, particularly QRS area, may provide additional insight into CRT-induced electrical changes beyond conventional ECG measures.

## Data Availability

The data underlying this article will be shared on reasonable request to the corresponding author.
